# Extraction of Biofilms From Ureteral Stents for Quantification and Cultivation-Dependent and -Independent Analyses

**DOI:** 10.3389/fmicb.2018.01470

**Published:** 2018-07-10

**Authors:** Matthias T. Buhmann, Dominik Abt, Stefanie Altenried, Patrick Rupper, Patrick Betschart, Valentin Zumstein, Katharina Maniura-Weber, Qun Ren

**Affiliations:** ^1^Laboratory for Biointerfaces, Empa, Swiss Federal Laboratories for Materials Science and Technology, St. Gallen, Switzerland; ^2^Department of Urology, Cantonal Hospital St. Gallen, St. Gallen, Switzerland; ^3^Laboratory for Advanced Fibers, Empa, Swiss Federal Laboratories for Materials Science and Technology, St. Gallen, Switzerland

**Keywords:** ureteral stent, catheter-associated urinary tract infection, biofilm, biomaterial-associated infection, crystalline biofilm, universal 16S qPCR

## Abstract

Ureteral stenting is a common surgical procedure, which is associated with a high morbidity and economic burden, but the knowledge on the link between biofilms on these stents, morbidity, and the impact of the involved microbiota is still limited. This is partially due to a lack of methods that allow for a controlled extraction of the biofilms from stents. Development of an appropriate *in vitro* model to assess prevention of biofilm formation by antimicrobial coatings and biomaterials requires a profound understanding of the biofilm composition, including the involved microbiota. This work describes an analytical pipeline for the extraction of native biofilms from ureteral stents for both cultivation-dependent and -independent analysis, involving a novel mechanical abrasion method of passing stent samples through a tapered pinhole. The efficiency of this novel method was evaluated by quantifying the removed biofilm mass, numbers of cultivable bacteria, calcium content, and microscopic stent analysis after biofilm removal using 30 clinical stent samples. Furthermore, the extraction of *in vitro* formed *Escherichia coli* biofilms was evaluated by universal 16S quantitative PCR, a cultivation-independent method to demonstrate efficient biofilm removal by the new approach. The novel method enables effective contamination-free extraction of the biofilms formed on ureteral stents and their subsequent quantification, and it represents a useful tool for comprehensive examinations of biofilms on ureteral stents.

## Introduction

The insertion of medical devices into the urinary tract, such as ureteral stents and urinary catheters, frequently causes irritations and urinary tract infections, which are the most common nosocomial infection (Warren, 2001). This is related to the encrustation with urine components that crystallize on the surface of biomaterials, and to the formation of microbial biofilms, complex communities of microorganisms embedded into a matrix of EPS. These encrustations and microbial biofilms (referred to as “biofilms” in the following) are proposed to be directly linked with patients’ discomfort and morbidity (Trautner and Darouiche, 2004). Different strategies to prevent biofilm formation on ureteral stents have been attempted, including the use of materials with antiadhesive properties, inhibiting the precipitation of urine components, and antimicrobials-releasing or -coated materials (Lo et al., 2014; El-Nahas et al., 2017). However, none of these strategies have led to clinical success to date. Accordingly, antimicrobial materials for urinary tract catheters are not used in clinical practice and not recommended by current clinical guidelines (Bonkat et al., 2017).

The lack of successful materials is partially due to the limited understanding of this particular type of biofilms and consequently, the lack of appropriate *in vitro* models that mimic the challenging *in vivo* conditions to guide the development of such materials (Buhmann et al., 2016). Previous work has mainly focused on extracting cultivable bacteria from stents via vortexing and sonication (Bonkat et al., 2011), by scraping with a scalpel (Paick et al., 2003), or by rolling stent sections on solid agar medium (Djeribi et al., 2012). While these methods allow for the detection of fast-growing urinary tract bacteria and provide acquisition of valuable qualitative or semi-quantitative information, they have clear limitations regarding biofilm quantification, reproducibility, and efficient extraction.

A method allowing for simultaneous cultivation-dependent and -independent quantification and analysis of microbiota, biofilm mass, and crystals still is missing. This lack of effective extraction methods might represent a major reason for the knowledge gap on the microbiota on ureteral stents, in contrast to the well-studied microbiota in urine or biofilms on Foley catheters (Frank et al., 2009; Whiteside et al., 2015). Stent biofilms frequently comprise different types of crystals, such as carbonate hydroxyapatite or calcium oxalate crystals that are held together by a cement of sticky biofilm EPS (Sighinolfi et al., 2015) and include complex deposits from human urine, such as DNA debris, protein, and metabolic products (Bouatra et al., 2013). Method development for a complete removal of these particular biofilms is challenging due to the rigid structure of the biofilms on the ureteral stent surface.

In this work, we describe a novel method, termed pinhole method (PM), for efficient extraction of native biofilms from ureteral stents for both cultivation-dependent and -independent analyses. We validated the method using *in vivo* clinical and *in vitro* model biofilms, and we demonstrated that the established biofilm extraction pipeline allows for efficient recovery of biofilm mass. Moreover, it avoids introduction of detectable DNA contamination, enabling cultivation-independent qPCR analysis. This work will facilitate quantitative microbiological, biochemical, and molecular analyses of biofilms from ureteral stents.

## Materials and Methods

### Study Material

A total of 30 ureteral stent samples from study patients (Percuflex Plus; Boston Scientific, Natick, MA, United States; 6F, 2 mm, 30 cm, sterilized with ethylene oxide) were surgically removed at the Urological Department of the Cantonal Hospital St. Gallen. The study was approved by the local ethics committee (EKSG 15/084). All procedures performed in the study were in accordance with the ethical standards of the institutional and national research committee, with the 1964 Helsinki Declaration, and its later amendments. Written informed consent was obtained from all individual participants included in the study. After surgical removal, the stents were rinsed in sterile saline solution, cut into two pieces of equal length and stored in 50 mL sterile centrifuge tubes containing 2 mL physiological saline solution at 4°C for a maximum of 5 days until analysis.

### Cultivation of Microorganisms

*Escherichia coli* U5/41 (DSM30083), a strain isolated from the urine of a patient with cystitis was obtained from the DSMZ culture collection (Kauffmann, 1944). The strain was maintained in glycerol stocks at -80°C and streaked out on solid tryptic soy broth medium (Sigma Aldrich) for experiments. Single colonies were picked to inoculate shaken fluid cultures in artificial urine medium (Brooks and Keevil, 1997) that were grown at 37°C overnight. *E. coli* biofilms were grown on halves of fresh ureteral stents using a drip-flow biofilm reactor comprising a peristaltic pump, 25 mL serological pipettes, silicone tubing, a T-junction, and a 0.2 μm PTFE syringe filter for pressure exchange (Supplementary Figure S1). Under constant flow of 750 mL/day artificial urine medium (Brooks and Keevil, 1997), stent halves were incubated in silicone tubing with an inner diameter of 3 mm, what refers to the average inner diameter of the human ureter (Zelenko et al., 2004).

### Manufacturing and DNA Decontamination of the Pinhole Device

Steel plates (30 mm × 100 mm × 1 mm, 1.44.04 stainless steel) were manufactured by first drilling a 1.9 mm hole with a CNC drill, followed by preparing the tapered shape with a 60° deburring tool. After preparing the tapered hole, the surface at the narrow side of the hole was polished using sandpaper (220 grit). Before usage, the pinhole devices were rinsed with 70% ethanol and wiped with paper tissue, followed by intense flame-sterilization for inactivation of DNA contamination.

### Extraction of Ureteral Stent Biofilms

**FIGURE 1:**
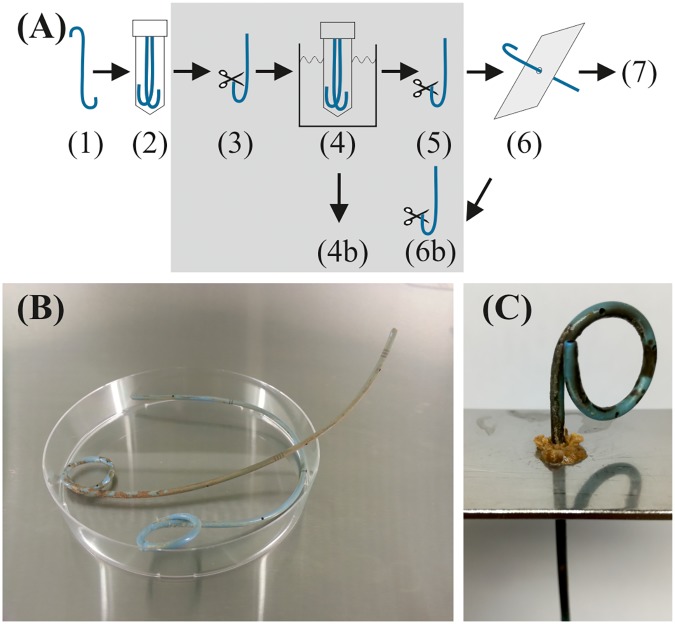
**(A)** Sequential extraction of stent biofilms for validation of the PM. Note that the steps in the gray box are only made for comparison with sonication and vortexing. (1) Stent removed from patient. (2) Stents were cut in the middle and stored in 50 mL polypropylene screw-cap tubes containing 2 mL saline solution. (3) Sampling of 2–3 mm sections of both loop tips by cutting with a scalpel for scanning electron microscopy before SV method. (4) Immersion in 50 mL saline, followed by 30 s vortexing, 60 s sonication, and 30 s vortexing. (4b) Centrifugation and collection of the pellet for biofilm mass weighing and bacterial growth assessment. (5) Sampling of the loop tips for SEM after SV. (6) PM extraction procedure. (6b) Sampling for SEM after PM. (7) Further analyses of the extracted biofilm. **(B)** Typical example of a ureteral stent with biofilm (cut into two parts). The biofilm (brown and whitish) is typically distributed unevenly. The diameter of the Petri dish is 80 mm, of the stent 2 mm. **(C)** Extraction of the biofilm using a tapered pinhole in a steel plate (SV had been performed before).

During all procedures, the stents were handled in a sterile work bench using aseptic techniques. Stents were handled exclusively with flame-sterilized forceps to avoid any introduction of DNA contamination, and relevant surfaces including gloves and pipettes were treated with a DNA-decontaminating agent (DNA-ExitusPlus, AppliChem). To validate the novel extraction method by comparison with sonication and vortexing (SV), the stents were subjected to a sequential extraction of the biofilms using SV first, followed by the PM, hypothesizing that the PM alone enables extraction of all material that would be released by SV (**Figure 1A**). The PM itself does not involve SV. For SV extraction, stent halves were submerged into 50 mL saline solution in a sterile 50 mL centrifuge tube, vortexed for 30 s, and placed in a sonication water bath (Bransonic 52, Branson Ultrasonics SA) at a frequency of 40 kHz and room temperature for 60 s, followed by another 30 s of vortexing, as described previously (Bonkat et al., 2012). The stent was removed for extraction with the PM, the sonication fluid was centrifuged at 7,500 × *g* for 20 min at 18°C and 48 mL of the supernatant were discarded. The pellet with SV-extracted material was resuspended in the remaining 2 mL and transferred to a balanced 2 mL microcentrifuge tube. After centrifugation for 5 min at 14,100 × *g*, the supernatant was removed and the pellet wet weight was determined using an analytical balance. Dry weight was determined after freeze-drying. After sampling for SEM by cutting 2–3 mm of the ureteral stent loop tips with a scalpel, each half of the stent was passed through the previously sterilized pinhole three times in each direction, using aseptic techniques. In between, the extracted biofilm was suspended in fractions of 2 mL saline solution, collected in a balanced 2 mL microcentrifuge, followed by centrifugation and determination of the pellet wet weight as before. As a negative control for sterility and abrasion of stent material, fresh, unused ureteral stents were extracted in three replicates.

### Microbiological Analyses

For a cultivation-dependent quantification of bacterial load, extracted biofilm pellets were each suspended in 500 μL physiological saline solution, followed by 30 s vortexing and 1 min ultrasound treatment in a sonication water bath. Serial dilutions were spotted in three replicates on 5% (v/v) sheep blood tryptic soy solid medium (Thermo Scientific) and incubated at 37°C in aerobic atmosphere overnight.

### Quantitative PCR

Cultivation-independent quantification of *E. coli* was performed via universal 16S qPCR as described previously (Nadkarni et al., 2002), using ultrapure water (DNase free water, Thermo Scientific), and ethylene oxide treated plastic ware (BiospherePlus, Sarstedt). Reactions were performed on a CFX96 thermocycler (Bio-Rad), using white 96-well PCR plates (Ref. no. 72.1981.232, Sarstedt), sealed with adhesive foil (Ref. no. 95.1999, Sarstedt). The reaction volumes were 15 μL, comprising TaqMan^®^ Environmental PCR Master Mix 2.0 (Applied Biosystems) with AmpliTaq Gold^TM^ DNA Polymerase UP, 300 nM HPLC-purified primers (forward primer 5′-TCCTACGGGAGGCAGCAGT-3′; reverse primer: 5′-GGACTACCAGGGTATCTAATCCTGTT-3′), 100 nM fluorescent probe (FAM-5′-CGTATTACCGCGGCTGCTGGCAC-3′-TAMRA), and 6 μL template DNA, generating a 465 bp amplicon. Cycle conditions were pre-heating 96°C for 1 s, initial denaturation 95°C for 10 min, followed by 55 cycles of 95°C for 15 s, and 62°C for 1 min (ramp rate of 1.4°C/s). The annealing temperature was optimized by gradient PCR and different primer/probe ratios were tested (*not shown*). Primer specificity was confirmed by agarose gel electrophoresis of PCR-amplified recDNA containing a single copy of the *E. coli* 16S rRNA gene (recDNA) and *E. coli* gDNA (*data not shown*).

*E. coli* was quantified via qPCR by amplification of 16S rRNA gene-containing DNA and correlating the determined quantity of 16S rRNA gene-containing DNA with CFU, which was obtained through an external standard curve (Supplementary Figure S2). Since the *E. coli* genome contains seven copies of the 16S rRNA gene, the determined number of 16S rRNA genes was divided by 7 (Klappenbach et al., 2001). Data analysis was performed with the LinRegPCR program version 2017.0 (Ruijter et al., 2009) and a one-point calibration method, employing an external recDNA standard of known copy number (Brankatschk et al., 2012). Details for cloning and data analysis are described in the Supplementary Methods section. DNA extraction from both *E. coli* cell suspensions and biofilms was performed using the Molysis^TM^ Complete5 kit (Molzym).

The limit of detection of the assay was defined according to the 3σ criterion of the IUPAC recommendations for analytical nomenclature as the mean of the lowest detectable concentration plus three times the standard deviation of the measurement from negative controls (Inczedy et al., 1998). Similarly, the limit of quantification was calculated using the 10σ criterion.

### Scanning Electron Microscopy (SEM)

For SEM, ureteral stents sections of 2–3 mm cut at the proximal and distal loop ends before and after SV treatment, and after the PM treatment, were fixed for 30 min in glutaraldehyde and formaldehyde as described elsewhere (Karnovsky, 1965) and stored in 0.9% saline solution at 4°C until dehydration using a series of increasing ethanol concentrations. Dry stent sections were treated with 1,1,1-trimethyl-*N*-(trimethylsilyl)silanamine and dried in a fume hood prior to sputtering with 7 nm Au/Pd in an EM ACE600 machine (Leica) and analyzed with an S-4800 SEM (Hitachi) at 2 kV acceleration voltage.

### X-ray Photoelectron Spectroscopy (XPS)

X-ray photoelectron spectroscopy measurements were performed with a Scanning XPS Microprobe (PHI VersaProbe II spectrometer, Physical Electronics) using monochromatic Al-Kα radiation (1486.6 eV) and a takeoff angle of 45° (with respect to the surface plane) as described previously (Rupper et al., 2017). The operating pressure of the analysis chamber was below 5 × 10^-7^ Pa during the measurements. For each sample, a randomly chosen spot in the middle of the stent surface (stent samples had been dried at 37°C) was analyzed using a microfocused X-ray beam with a diameter of 100 μm. Dual beam charge compensation was used to compensate sample charging. Survey scan spectra (0–1,100 eV) were acquired with an energy step width of 0.8 eV, an acquisition time of 160 ms per data point and pass energy of 187.85 eV. Higher resolution narrow spectra over carbon C 1 s and oxygen O 1 s regions were acquired with an energy step width of 0.125 eV, an acquisition time of 1.9 s (carbon) and 1.2 s (oxygen) per data point and a pass energy of 29.35 eV. The binding energy is referenced to the aliphatic carbon at 285 eV. Data treatment and peak-fitting procedures were performed using the CasaXPS software version 2.3.16. For quantification, PHI sensitivity factors were corrected for our system’s transmission function and spectrometer geometry.

### Inductively Coupled Plasma Optical Emission Spectrometry (ICP-OES)

Extracted biofilms were freeze-dried in 2 mL polypropylene tubes, followed by determination of the dry biofilm mass. Hydrolysis was performed by adding consecutively two times 100 μL of concentrated nitric acid. The tubes were filled with 200 or 400 μL deionized water, heated within 5 min to 120°C, extracted for 8 min at 2 × 10^6^ Pa in a turboWAVE microwave oven (MLS) and subsequently diluted to 2 mL with deionized water. The calcium containing solutions were injected into an Optima 3000 instrument (PerkinElmer) and optically analyzed at 422.673 nm. The instrument was calibrated using calcium chloride standard solutions.

### Statistical Analyses

*T*-test analyses were performed using the Origin 2017G software package (OriginLab Corporation). Standard deviations are signed as “±.” Pearson’s correlation was calculated with GraphPad Prism version 6.07 (GraphPad Software).

## Results and Discussion

### A Novel Abrasion-Based Biofilm Extraction Method

Biofilms from ureteral stents were effectively extracted by repetitively passing the ureteral stent through a tapered pinhole in a steel plate with a slightly smaller diameter than the stent (i.e., 1.9 and 2.0 mm, respectively). To validate the PM in a setting with realistic mechanical properties of the specimen, extraction of *in vivo* biofilms was performed from clinical ureteral stents (indwelling time 3 weeks to 5 months). A previously reported method of SV (Bonkat et al., 2011) was used for direct comparison.

The evident uneven distribution of the biofilms on ureteral stents (**Figures 1B,C**) made it impossible to divide one stent into sections and to compare technical replicates. Thus, the complete biofilm of each stent was extracted sequentially by SV first, followed by the PM (**Figure 1A**). The sequential extraction allowed comparison of the combination of PM and SV with SV alone, therefore, in the following, “PM” denotes the extracted biofilm that was extracted via SV followed by the PM. In practice the PM alone is sufficient to extract the biofilm from the stent surface, which includes the fraction that can be removed by the SV treatment alone, and an additional fraction which can only be removed by the increased mechanical force of the PM treatment. The extraction of several stent samples (with similar indwelling time) with the PM alone revealed microscopic appearance similar to SV plus PM, indicating a quantitative extraction (*data not shown*).

### Gravimetric Analysis Reveals Increased Biofilm Removal by the PM

**FIGURE 2:**
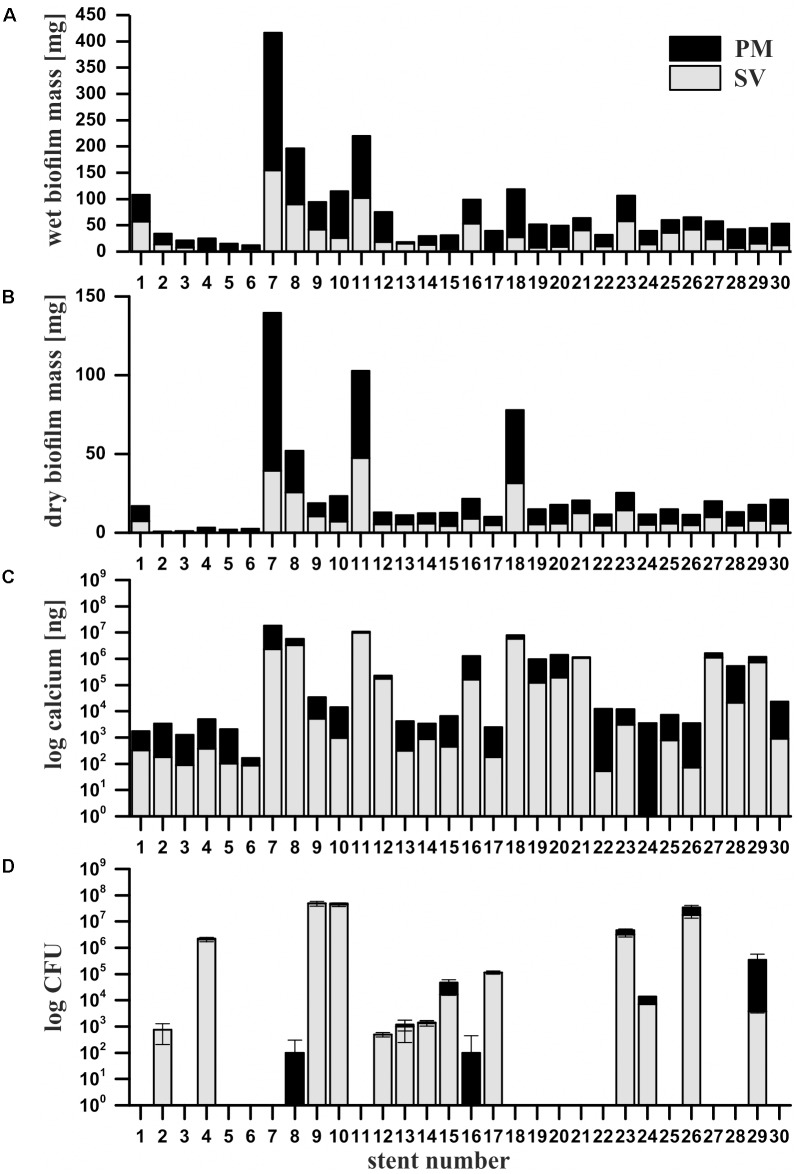
Quantification of wet **(A)** and dry **(B)** biofilm mass, calcium contents **(C)** and cultivable microorganisms **(D)** from extracted clinical ureteral stent biofilms. The stents were sequentially extracted with the sonication and vortexing method (SV, gray bars), followed by the mechanical abrasion with a pinhole. Data from the PM reflect the combination of PM and SV. Black bars represent the additional biofilm mass, calcium and CFU yielded with the PM. Error bars represent the standard deviation between three technical replicates.

For all samples, the PM was able to remove additional biofilm that was not extracted by SV alone, as quantified by weight of the extracted wet and dry biofilm mass (additional 36 ± 18% of wet and 40 ± 17% of dry biofilm mass, *n* = 30, *p* < 0.05, Student’s *t*-test, **Figures 2A,B**, and Supplementary Figures S3A,B). Since the PM additionally removed small quantities of the stent coating (9.4 ± 1.9 mg, *n* = 3), as determined by extracting non-used stents under the same wet conditions, this amount was subtracted from the wet biofilm mass. In a dry state, the weight of the removed coating was too small to be considered (i.e., ≤ 0.4 mg). The PM-extracted freeze-dried biofilm mass correlated well with the wet biofilm mass (**Figures 2A,B**) [Pearson *r* = 0.9482, 95% CI 0.8930–0.9753, *r*^2^ = 0.8991, *p* (two-tailed) < 0.0001, *n* = 30], therefore, wet weight can be used as an approximate for the overall biofilm mass.

It has to be noted that the novel method does not provide extraction of biofilm deposited on the stent lumen. Laube et al. (2008) demonstrated that most (i.e., 75%) of the ureteral stents which seem to be obstructed according to failure of the Seldinger technique (i.e., the failure to introduce a guide wire into the stent lumen), do not show significant intraluminal deposits and, therefore, the biofilm in the inner part of the stent can be regarded as negligible. Furthermore, the luminal biofilm is not in direct contact with the urothelium and rarely responsible for stent dysfunction as the main urine flow occurs outside the ureteral stent (Yossepowitch et al., 2001). This is in contrast to Foley catheters, where the main urine flow is clearly inside the catheter and the biofilm formed inside the catheter is regarded as the major problem, even though biofilm on the catheter surface can lead to damage of the urethral mucosa and hematuria resulting from catheter removal (Stickler and Feneley, 2010). We attempted to isolate the luminal biofilm by punching through the stent with a steel wire. However, this approach cannot be standardized or be performed under sterile conditions. Nevertheless, compared with the previously reported SV method the PM allowed higher biofilm mass removal from stents, demonstrating its potential for clinical studies on the ureteral stent biofilms.

### Calcium Analysis and XPS Confirm the Effectiveness of the Abrasion-Based Extraction

Calcium is commonly present in mineral precipitates found on ureteral stents (Uvarov et al., 2011). Complementary to biofilm mass quantification, calcium extracted from the stent samples was quantified via ICP-OES. Similar to the biofilm mass, the PM yielded additional amounts of extracted calcium compared to SV alone (*p* < 0.05, Student’s *t*-test, **Figure 2C**, and Supplementary Figure S3C). To exclude a potential contamination with calcium abraded from the stent material, calcium contents of extracts from non-used control stents treated with SV and subsequent PM were determined and subtracted accordingly (i.e., 0.233 ± 0.004 μg for SV and 0.577 ± 0.210 μg for the PM). The calcium content significantly correlated with the total biofilm mass, even though the number of clinical ureteral stents was limited to 30 (Pearson *r* = 0.9162, 95% CI 0.8298–0.9597, *r*^2^ = 0.8394, *p* < 0.0001, *n* = 30).

Additionally, the surface chemistry of a non-used ureteral stent before and after the extraction (non-treated, SV and PM) was compared by XPS analysis. The spectra of the non-treated stent and the SV-treated stent were very similar, indicating that the coating did not get released during the SV treatment. After treatment with the PM, additional signals likely representing functional groups of the polymeric stent material were observed, indicating that the PM removed some of the coating material (see Supplementary Figure S4 and Supplementary Table S1). Yet, these signals were weak and corresponded to negligible amount of mass (as determined gravimetrically 9.4 ± 1.9 mg). The analysis of used ureteral stents after extraction with the PM showed that no calcium was detected in any of the samples, indicating the removal of all crystalline components (Supplementary Table S1).

### Cultivation and SEM Imaging Reveal Increased Extraction of Bacteria by the PM

**FIGURE 3:**
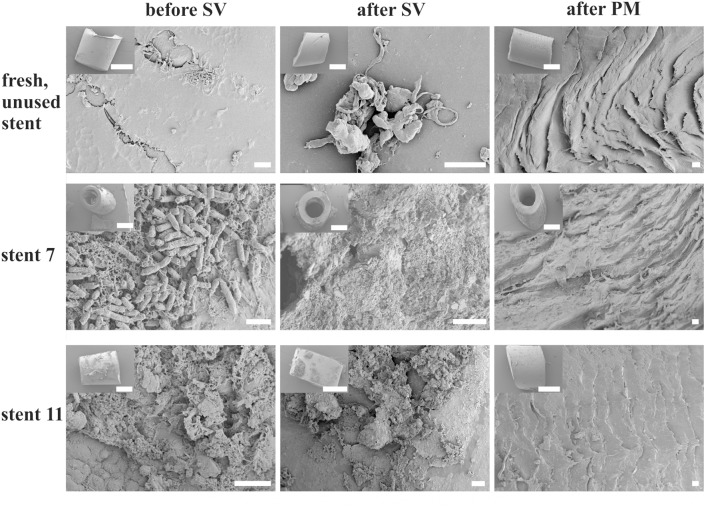
Scanning electron micrographs of the surface of ureteral stent sections indicate efficient removal of the biofilms by the PM. Exemplary sections of stent 7, 11, and an unused control stent prepared before sonication and vortexing (before SV), afterward (after SV), and after extraction with the pinhole method (after PM). The stent material was roughened by the PM, but biofilms were removed seemingly completely. Scale bars equal 10 μm. Inserts: overview on the imaged sections, bars = 1 mm.

In contrast to the significantly increased amounts of extracted biofilm mass and calcium yielded by the PM, the number of extracted cultivable microorganisms, as determined by dilution plating and quantified as CFU, increased only slightly with the PM (**Figure 2D**). Remarkably, in a small number of samples (i.e., samples No. 8 and 16) only the PM enabled the extraction of cultivable bacteria. This could be caused by sonication which killed certain sensitive bacteria, or due to a “hidden” localization of the bacteria in deeper layers of the biofilm that cannot be removed by sonication (Oliva et al., 2015). Limitations of both, cultivation and SEM imaging, became obvious since SEM imaging occasionally revealed microorganisms within a sample (e.g., No. 11) (**Figure 3**) for which no bacterial growth was observed (**Figure 2D**). On the contrary, in some cultivation-positive samples only few or no bacteria were visible on SEM images. This might be explained either by a patchy growth of bacteria only on certain sections of the stents, or by the possibility that the bacteria are covered by layers of extracellular polymers and mineral precipitates. Overall, these results demonstrate the need for an extraction method which allows sensitive analysis of the biofilm derived from the complete device. Furthermore, it has to be considered that the success of cultivating bacterial species from the urinary tract highly depends on the cultivation conditions, since certain species require anaerobic cultivation or prolonged incubation time (Hilt et al., 2014). In addition, our data show no correlation between cultivable bacteria and the amount of biofilm mass (**Figures 2A,B,D**), presumably due to large amounts of inorganic components. For example, samples 2 and 4 showed high numbers of CFUs, while the overall stent biofilm mass was very low.

### Cultivation-Independent Assessment of Bacteria via 16S qPCR Confirms Increased Recovery of Ureteral Stent Biofilms by the Novel PM

To compare the biofilm extraction efficacy by PM and SV for the subsequent identification and quantification of microbes using a cultivation-independent method, we cultivated *E. coli* biofilms on halves of ureteral stents in a drip-flow biofilm reactor under continuous flow of artificial urine medium (Supplementary Figure S1), and assessed *E. coli* cell numbers via both dilution plating and a 16S qPCR assay (Nadkarni et al., 2002). Even though the applied growth system allowed formation of *E. coli* biofilms, it cannot be regarded at this stage as a predictive *in vitro* biofilm model for the assessment of ureteral stent biomaterials due to non-optimized growth media, reactor surfaces, bacterial communities, etc. Further optimization is under investigation, however, beyond the scope of this study.

Under our experimental conditions the qPCR assay exhibited a linear operative range over 6 log units from 9 × 10^8^ to 7 × 10^3^ *E. coli* CFU equivalents (*r*^2^ = 0.993) with a limit of detection at around 550 *E. coli* CFU equivalents, and a lower limit of quantification at 1400 *E. coli* CFU equivalents (Supplementary Figure S2). The ureteral stents were covered by biofilms in amounts comparable to the clinical samples (30–84 mg/stent). Similar to the extraction of clinical samples, the PM removed additional amounts of biofilm mass than SV alone (Supplementary Figure S3D, Student’s *t*-test, *p* < 0.05). In accordance, the number of extracted *E. coli* cells was increased when using the PM, as assessed via dilution plating and qPCR, by 0.8–11.9% CFU and 1.7–12.7% CFU equivalents, respectively (**Table 1**). Overall, assessment of *E. coli* via dilution plating correlated with qPCR [Pearson *r* = 0.691, 95% CI 0.1950–0.9059, *r*^2^ = 0.4782, *p* (two-tailed) = 0.0127, *n* = 12] (Supplementary Figure S5). Furthermore, our results show that the PM avoided contamination of the biofilms during extraction with bacterial DNA (Supplementary Material qPCR controls). A quantitative extraction and detection of the last missing portion of biofilm microbiota may not be relevant for clinical microbiology diagnostics, but it can be critical for sequencing-based microbiome profiling of samples with low bacterial load, in order to avoid a dominance of contaminating DNA in sequence libraries (Mühl et al., 2010; Laurence et al., 2014; Salter et al., 2014).

**Table 1:** Additional biofilm mass and amounts of bacteria after extraction with PM compared to SV.

Stent replicate	% Additional CFU equivalents (qPCR)	% Additonal CFU	% Additional biofilm mass
1	12.7	1.6	20.9
2	3.6	11.9	38.1
3	5.8	5.6	18.2
4	8.1	3.8	25.7
5	1.7	0.8	19.5
6	2.1	3.4	25.5


*CFUs, colony forming units; CFU equivalents, CFU assessed via qPCR.*

In the analysis of the efficacy of antimicrobial coatings and materials mostly cultivation-dependent methods have been used, even though depending on the selected cultivation-dependent method, high variations in antimicrobial efficacy can be obtained for the same material (van de Lagemaat et al., 2017). For example, mechanical removal of surface-attached bacteria can potentially lead to release of the antimicrobials, making cultivation-dependent analysis unreliable. Therefore, cultivation-independent assessment of the bacterial load via qPCR is valuable for the conclusive analysis of the efficacy of antimicrobial coatings and materials.

Plotting CFU against CFU equivalent, as obtained via qPCR, showed some variation for cultivated *E. coli* biofilms (Supplementary Figure S5), while a strong correlation between CFU and 16S rRNA gene copy number for exponentially growing *E. coli* suspension cultures was observed (Supplementary Figure S2). This may be explained by the presence of varying amounts of extracellular DNA, non-viable, or viable but not cultivable bacteria in the biofilms (Fux et al., 2005; Flemming et al., 2016), caused by small variation such as shear force, or gradients in nutrient supply between the replicates.

## Conclusion

This work introduces a fast and reproducible method for extraction of the total native biofilm from the surface of ureteral stents, allowing for exact quantitative, cultivation-dependent and -independent analyses at the same time. Compared to SV, the PM yielded significantly higher amounts of extracted biofilm mass and minerals, presumably due to the rigid mechanical properties of urinary biofilms. Further, the PM can be standardized, and therefore allows quantification of the extracted biofilm, in contrast to simple scraping the biofilms off with a scalpel (Paick et al., 2003). Even though, compared to SV, the PM provided only slightly but constantly increased recovery of bacteria from stent biofilms, the DNA contamination free handling makes it useful for cultivation-independent, PCR-based microbiome profiling. In summary, the PM enables a spectrum of novel analyses that may help to understand the correlation between patients’ morbidity and stent biofilm formation, and has already proven to be expedient within ongoing clinical trials (NCT02845726, NCT02871609).

## Author Contributions

MB, QR, and DA designed the study. MB and QR conceived the experiments. MB and SA conducted the experiments. PR performed XPS analyses. MB analyzed the results and drafted the manuscript. MB, DA, and QR wrote the manuscript. DA, PB, VZ, and KM-W gave scientific input to the study. All authors reviewed and approved the manuscript.

## Conflict of Interest Statement

The authors declare that the research was conducted in the absence of any commercial or financial relationships that could be construed as a potential conflict of interest.

## Abbreviations

bp: base pair
CFUs: colony forming units
EPS: extracellular polymeric substances
gDNA: genomic DNA
ICP-OES: inductively coupled plasma optical emission spectrometry
PM: pinhole method
qPCR: quantitative PCR
recDNA: recombinant DNA
SEM: scanning electron microscopy
SV: sonication and vortexing
XPS: X-ray photoelectron spectroscopy
