# Supplementary material for: Extraction of Biofilms From Ureteral Stents for Quantification and Cultivation-Dependent and -Independent Analyses
Source: Front Microbiol. 2018 Jul 10;9:1470. doi: 10.3389/fmicb.2018.01470 (PMC6052902; doi:10.3389/fmicb.2018.01470)
Supplement: Supplementary file 1 [file Data_Sheet_1.pdf]

Supplementary Material

Extraction of Biofilms from Ureteral Stents for Quantification and Cultivation –Dependent and -Independent Analyses

Matthias T. Buhmann*, Dominik Abt, Stefanie Altenried, Patrick Rupper, Patrick Betschart, Valentin Zumstein, Katharina Maniura-Weber, Qun Ren*

*** Correspondence:**
Matthias.Buhmann@empa.ch
Qun.Ren@empa.ch

1.1 Supplementary Figures

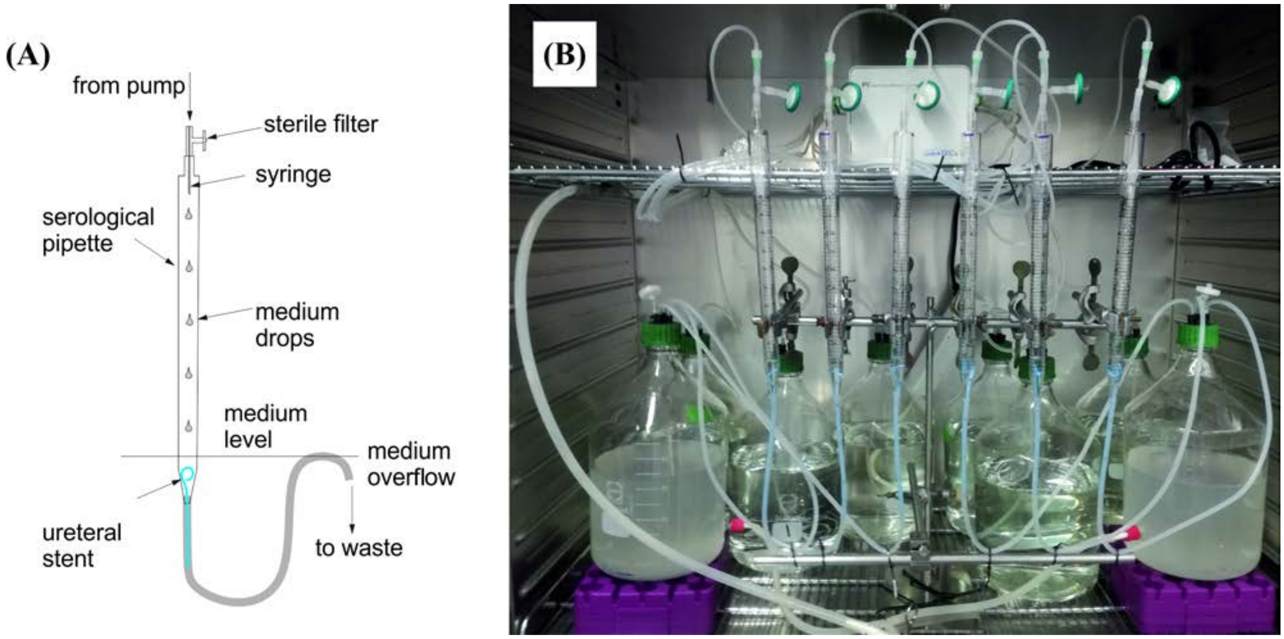

Figure S1. Drip-flow biofilm reactor for the cultivation of biofilms on ureteral stent material. **(A)** Schematic drawing of an individual reactor for growing biofilms on half of a ureteral stent. **(B)** Overview of the 6 replicates in an incubator.

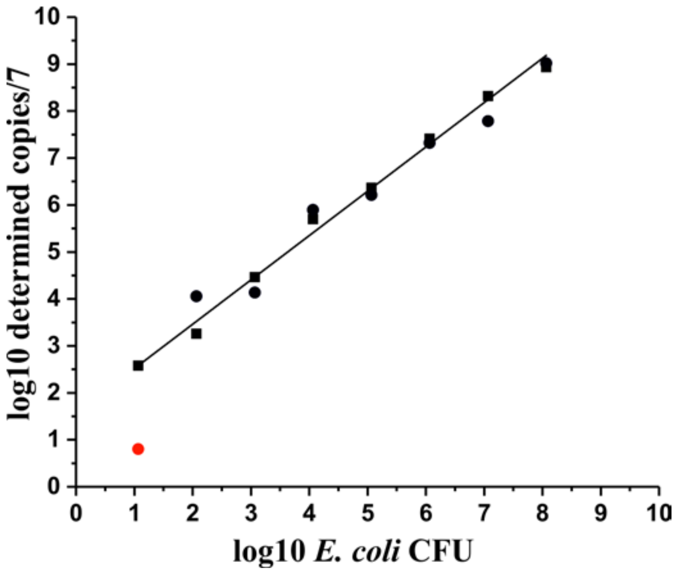

Figure S2. Standard curve with gDNA for the 16S qPCR assay. 16S gene copy number as determined via qPCR (divided by 7 copies per *E. coli* genome) plotted against CFU derived from the culture used for DNA extraction (slope = 0.94 ± 0.02, intercept 1.57 ± 0.15, $r^2 = 0.993$). The limit of detection was at around 550 *E. coli* CFU equivalents and the lower limit of quantification at 1400 *E. coli* CFU equivalents. Each dilution was analyzed in technical replicates (circles and boxes), an outlier excluded from linear regression is marked red.

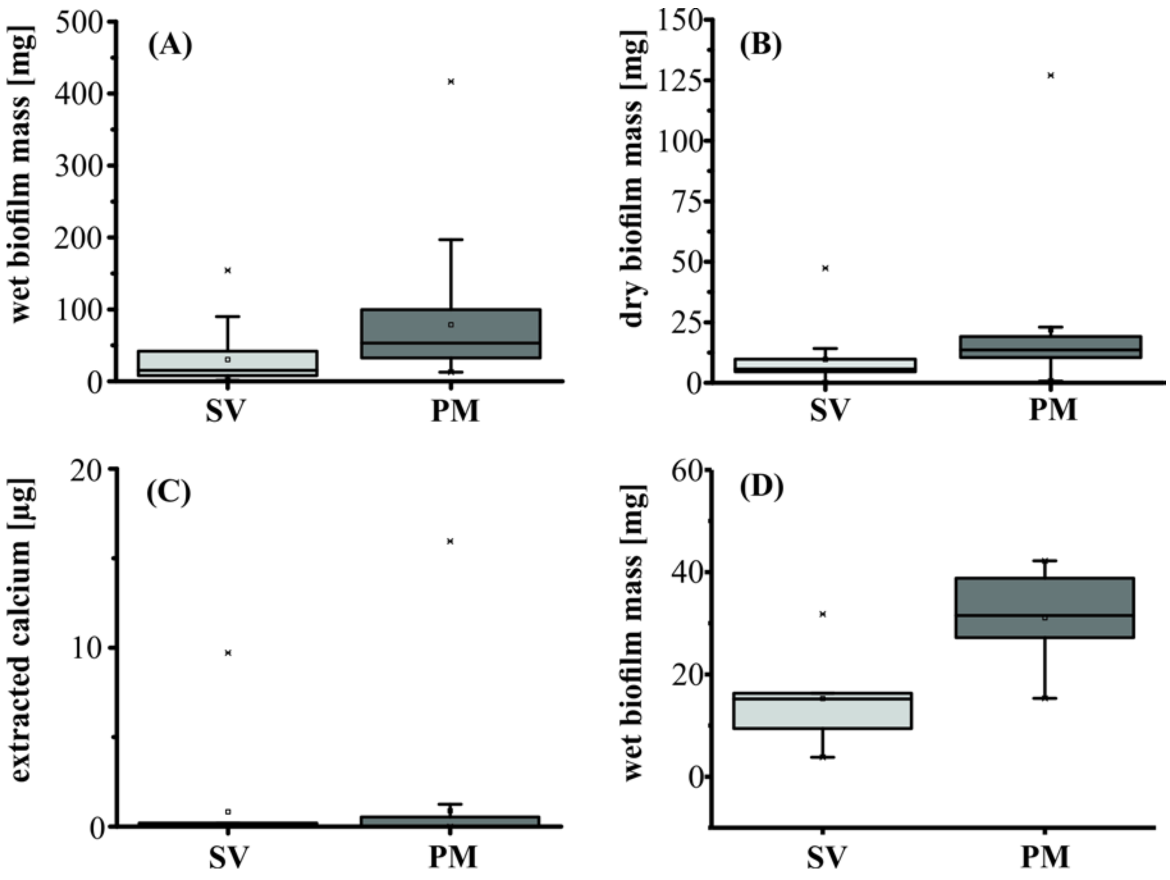

Figure S3. Increased recovery of biofilm mass and calcium from *in vivo* and *in vitro* biofilms. The PM (SV + PM) allowed significantly increased recovery extracted wet **(A)** and dry **(B)** biofilm mass, as well as calcium **(C)** from *in vivo* biofilms than SV alone (Student's *t*-Test, one sample, $n = 30$, $p < 0.05$). Similarly, significantly higher amounts of biofilm mass were extracted from *in vitro* formed *E. coli* biofilms using the PM **(D)**. The box represents 25 and 75%, with the median as a line. The whiskers indicate outliers (coefficient 1.5). 99% and 1% are represented by "X", the mean by a square, minimum and maximum by straight lines.

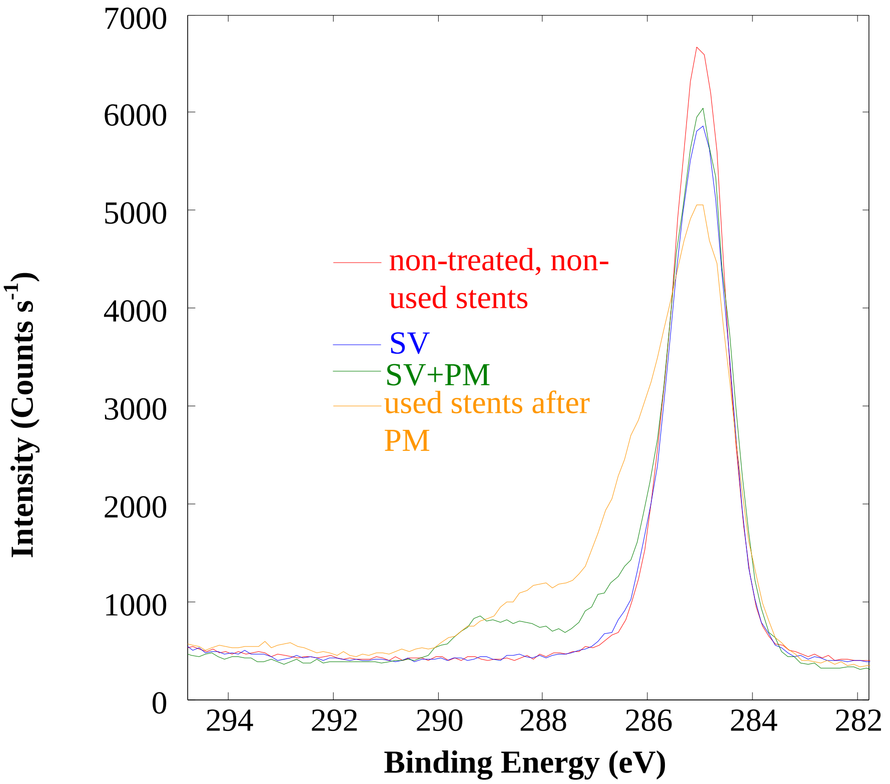

Figure S4. Comparison of high-resolution C 1s photoelectron spectra (XPS) between non-used ureteral stents before (non-treated) and after (SV, SV+PM) extraction with used stents after PM extraction.

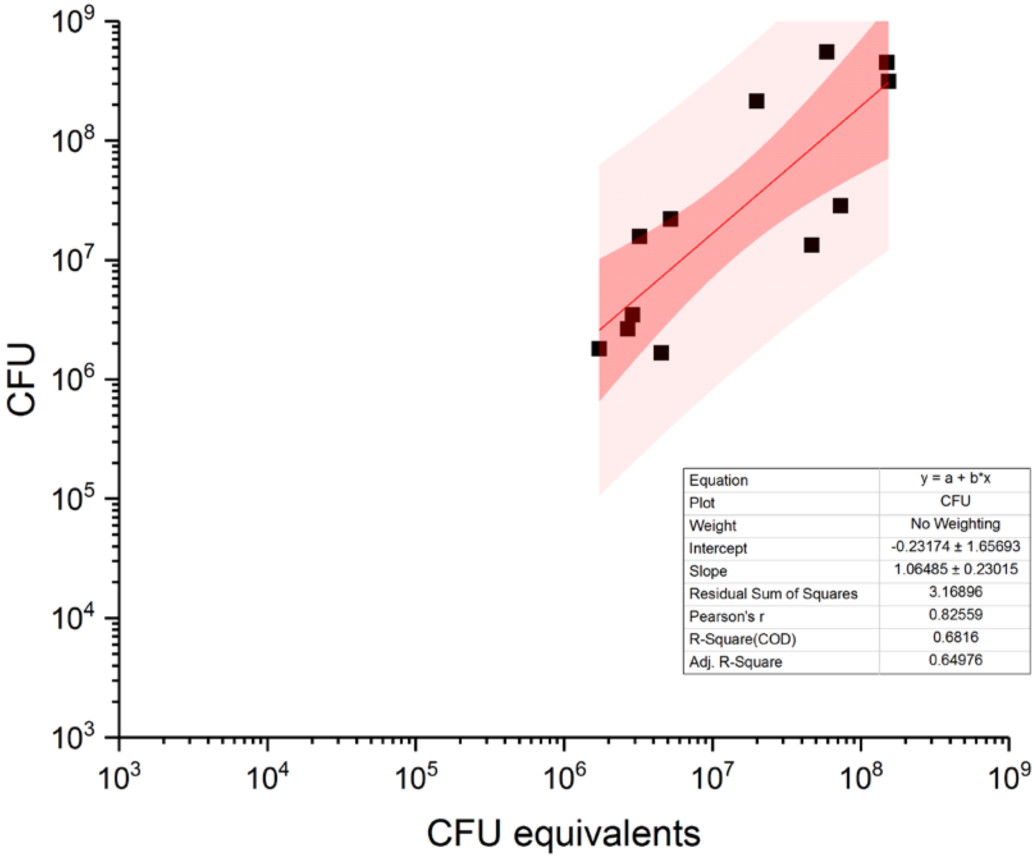

Figure S5. Correlation of CFU and CFU equivalents, as determined via qPCR for *E. coli* biofilms. The graph represents a linear fit for data points from qPCR and dilution plating for SV and PM (alone) extracted biofilms. The dark red band represents 95% confidence band, the light red band the 95% prediction band.

1.2 Supplementary Tables

Table S1: Surface elemental composition (atomic % concentrations) of different samples determined from XPS measurements.

atomic %	C	O	N	Si	Na	Cl
non-treated	52.3	25.1	-	22.6	-	-
SV	52.4	26.1	-	21.5	-	-
SV+PM	60.3	27.2	-	9.2	1.8	1.5
used stents after PM (average ST009/ST016)	62.3	21.5	6.4	6.3	1.3	2.2

1.3 Supplementary Methods

1.3.1 Generation of a recombinant 16S rDNA standard for the 16S qPCR assay

For the generation of a plasmid standard, a 465 bp amplicon of the 16S gene was PCR-amplified from *E. coli* DSMZ 30083 gDNA, using the primer set described above (300 nM), proof-reading blunt-end generating DNA polymerase (Phusion, using Phusion GC buffer, Thermo Scientific) with 6 µL template in a final volume of 15 µL and PCR conditions identical with the qPCR. Amplicons were purified on a 1% agarose gel, extracted (geneJet, Thermo Scientific) and ligated into a pJet 1.2/blunt cloning vector (Thermo Scientific) using T4 DNA ligase. The vector was transformed into *E. coli* dH5 alpha via heat shock (30 min 4 °C, 2 min 42 °C, 2 min 4 °C), followed by recovery in SOC medium (45 min, 37 °C, 60 rpm), spreading on selective LB solid medium containing 100 µg mL^{-1} ampicillin (LB/Amp) and incubation at 37 °C overnight. Ligation resulted in 488 colonies compared to 3 colonies for the empty vector. 5 colonies were picked for miniprep (zymoPURE, Zymo Research) and sequenced with pJet1.2-forward and reverse primers. A single clone was selected for generation of the standard and amplified in a 50 mL fluid culture (LB/Amp) for subsequent midiprep (CompactPrep, Qiagen). The recDNA was eluted into DNAse-free water (Thermo Scientific) and quantified with a spectrophotometer.

1.3.2 qPCR data analysis

Non-baseline corrected fluorescence raw data were exported from the thermocycler software (CFX manager v. 3.1, Bio-Rad) into Excel and imported into the LinRegPCR program (v. 2017.0) for determination of the window of linearity, baseline and primer efficiency via fluorescence increase (Ruijter et al., 2009). The estimated initial template copy numbers (N_0) for each reaction, with N_t as fluorescence threshold, PCR efficiency E and the number of cycles until reaching the threshold C_t was calculated as described previously (Equation 1) (Ruijter et al., 2009).

$$N_0 = \frac{N_t}{E^{CT}} \text{ (Equation 1)}$$

All samples of one qPCR run were treated as one amplicon group for setting of a common window of linearity. Only in exceptions, window of linearity and florescence threshold were adjusted manual. Potential variations in primer efficiency between different templates were corrected using the one-point calibration method according to (Equation 2), using an external recDNA standard of known number (Brankatschk et al., 2012). Here, mean PCR efficiencies as calculated by the LinRegPCR software were used for $E_{standard}$ to equal out small variations for the calibrator. The corresponding copy number was calculated from the DNA concentration, Avogadro's constant and the molecular weight of 2124727.9 g mol^{-1} of the 3439 bp plasmid with 16S gene insert.

$$N_{0\,Sample} = N_{0\,Standard} \times \frac{E_{Standard}{}^{CT_{Standard}}}{E_{Sample}{}^{CT_{Sample}}} \text{ (Equation 2)}$$

1.4 qPCR controls

Since the qPCR assay also detects DNA from a broad range of bacteria different from *E. coli*, absence of amplification indicates absence of bacterial DNA. The extraction of empty, non-used blank stents revealed no amplification for 5 out of 6 replicates, for the single amplified replicate below the limit of detection similar to the no template control (3 out of 6 amplified, all below limit of detection). Addition of recDNA to the reaction as positive control revealed absence of PCR inhibitors in the abraded material (*not shown*).
